# Danish Prostate Registry (DanProst) – an Updated Version of the Danish Prostate Cancer Registry, Methodology, and Early Results

**DOI:** 10.1007/s10916-023-01991-8

**Published:** 2023-09-13

**Authors:** Hein Vincent Stroomberg, S. Benzon Larsen, G. Samsø Lanthén, T. Kjaer Nielsen, J. T. Helgstrand, K Brasso, A Røder

**Affiliations:** 1 Copenhagen Prostate Cancer Center, Department of Urology, Copenhagen University Hospital – Rigshospitalet, Ole Maaløes Vej 24, 7521, Copenhagen, DK-2200 Denmark; 2 Section of Biostatistics, Department of Public Health, University of Copenhagen, Copenhagen, Denmark; 3 Survivorship and Inequality in Cancer, Danish Cancer Society Research Centre, Copenhagen, Denmark; 4 Section of Epidemiology, Department of Public Health, University of Copenhagen, Copenhagen, Denmark; 5 Department of Clinical Medicine, University of Copenhagen, Copenhagen, Denmark

**Keywords:** Registry, DanProst, DaPCaR, Prostate, Prostate cancer, Denmark

## Abstract

In 2016, we introduced the Danish Prostate Cancer Registry (DaPCaR) which was built on the National Pathology Register from 1995 to 2011. DaPCaR was laborious to use as most data had to be manually imputed with no regular updates. In here we present a new comprehensive centralized prostate registry called the Danish Prostate Registry (DanProst), which includes all men having undergone any histological evaluation of prostate tissue merged with laboratory-, treatment-, prescription data as well as vital status. Here the data included and the methodology of DanProst are described. DanProst is built upon all men with a histological assessment of the prostate from the Danish National Registry for Pathology. The primary histology and potential prostate cancer histological diagnosis for each unique individual is extracted and translated by newly made algorithms for topography, procedure, diagnostic conclusion, and pathological staging. Further information is added from DaPCaR, the CPR Registry, the Danish Cause of Death Registry, the Danish Cancer Registry, the National Patient Registry, the Danish Register of Laboratory Results for Research, and the Danish National Prescription Registry. The translation algorithms were validated based on the comparison with DaPCaR in the period 2010–2016. DanProst includes 190,422 men. A total of 95,152 (50%) men are diagnosed with prostate cancer until 2021. Median diagnostic PSA was 11 ng/ml, most men are diagnosed by ultrasound-guided biopsy (N = 63,751; 67%), and most frequently defined primary treatment was radical prostatectomy (N = 14,778; 19%). DanProst to DaPCaR coherency was > 99%, 95%, and 94% for the primary histological procedure, primary histological conclusion, and diagnostic histological conclusion, respectively. DanProst is a continuously updated, centrally kept, validated registry with automatic integration of data from other national registries, allowing for contemporary nationwide analysis in men with histological assessment of the prostate.

**Supplementary Information:**

The online version contains supplementary material available at 10.1007/s10916-023-01991-8.

## Introduction

Denmark has a long history of highly valid health registries, including the world’s oldest cancer registry dating back to 1947 [1]. These registries can be linked through a unique identification number (CPR), given to all residents either at birth or immigration, which allows for epidemiological research across all medical disciplines [2]. In 2016, we published the method for a cross-linked registry, The Danish Prostate Cancer Registry (DaPCaR) built on the National Pathology Register from 1995 to 2011, to address outcomes associated with prostate cancer diagnosis and treatment [3]. Following an update in 2018, the most recent version of DaPCaR includes a population of 153,373 unique men with a histological assessment of the prostate from 1995 to 2016. So far, this registry has resulted in several publications describing different epidemiological aspects of prostate cancer diagnosis and outcomes [4–13]. The two versions of DaPCaR were laborious to use as most data had to be manually imputed with no regular updates. Meanwhile, Danish Health Authorities were working on a novel online platform where all Danish health registries could be accessed, following regulatory approval, to facilitate easier access to data, including day-to-day updates of all registries. The platform is termed “The Research Machine” (Forskermaskinen) and is administered by the Danish Health Data Authority which is part of The Ministry of Health.

We believe it is of interest to create a new comprehensive prostate registry, including all men having undergone any histological evaluation of prostate tissue, and merge this with all available health data including laboratory-, treatment-, prescription data as well as vital status. Using knowledge and codes from the original DaPCaR we here describe the methodology and initial results of the new Danish Prostate Registry (DanProst) embedded at Forskermaskinen. This paper aims to describe the data included and the methodology of code combination and discuss the opportunities of DanProst. A secondary aim is that the translational algorithms can be used as a template for other projects that want to utilize, Systematized Nomenclature of Medicine (SNOMED), hospital system classification system (SKS), nomenclature properties and units (NPU), and international classification of diseases (ICD-10) nomenclature to extract information for research and create integrated registries.

## Methods

DanProst is a new registry embedded at Forskermaskinen. The registry enables day-to-day updates, and easy access to data, and includes linkage between Danish health registries for research purposes. The registry replaces our previous DaPCaR [3], and includes all men with any histological investigation of prostate tissue in Denmark between 1995 and 2021.

The primary source of information in DanProst is retrieved from the Danish National Registry for Pathology. The Danish National Registry for Pathology contains information on all histological assessments and diagnoses performed by pathologists in the Danish healthcare system, including in private practices from 1997 and onwards [14]. The register has nationwide coverage and is updated daily. The registry reports data in the form of SNOMED codes. From the Danish National Registry for Pathology information on all men with a histological assessment containing the SNOMED code for the prostate (“T77*”, with * depicting any or no character following) is extracted. Including both the primary prostate assessments and information on any subsequent assessments. The database currently contains 288,499 unique histological assessments. Each assessment holds information on CPR numbers, date of assessment, the department of pathologist, and histopathological assessment of the tissue including diagnoses and descriptions.

From all unique histological assessments, the primary histology for each unique individual is extracted. The updated initial version of the database contained 182,769 individuals with at least one histological assessment. The SNOMED codes related to these histological assessments are translated by specifically made algorithms (Supplementary Fig. 1 to 8). In short, there is an algorithm for topography, procedure, diagnosis, pathological tumor lymph node and metastasis (TNM) staging, radical prostatectomy, number of biopsies, and the number of positive biopsies. Specifically, the number of prostate biopsies is defined by counting the number of occurrences of the P309*, P31071, P31069, P3106[A|B], or P32103 SNOMED codes in one histological grading. Similarly, the number of positive prostate biopsy cores is defined by counting the occurrences of M8140[2–9|X] or M8231* SNOMED codes in one histological grading. Pathological departments of assessment are translated utilizing the healthcare system organization registration (SOR) codes.

Primary histological assessments from 1995 to 1997 are added from DaPCaR. DaPCaR contains all men with a prostate histological assessment from 1995 to 2016 [3]. Moreover, DaPCaR holds prostate cancer-specific diagnosis manually assessed if information was missing from the registry. It also holds manually obtained prostate-specific antigen (PSA) measurements for a part of the men included.

For simplicity, only the first histology assessment and the first histology with a prostate cancer diagnosis are kept in the base DanProst, yet all translational algorithms can be used for follow-up histological assessments as the SNOMED coding is universal. Utilizing the CPR Registry personal information, such as date of birth, is added to DanProst [15]. The CPR number from the CPR registry also functions as a key through which DanProst retrieves information from all other registries. Vital/emigration status is not included in the base dataset as information on vital status and cause of death can be dynamically added based on the requirement of a specific study. The cause-specific vital status can be extracted from the Danish Cause of Death Registry [16]. It is obligatory in Denmark for a physician who declares a person dead to fill in a death certificate. This includes the direct underlining cause as well as other diagnoses that could contribute to the cause of death.

The histological diagnosis of prostate cancer is added to DanProst by assessing all unique histological assessments after translation by the diagnostic algorithm (Supplementary Fig. 3). Only the first diagnosis of prostate cancer was kept for each unique man. Diagnostic TNM staging was extracted from the Danish Cancer Registry if the date of diagnosis was registered within a year of the date of diagnosis in DanProst [1]. Concerning prostate cancer, tumor characteristics were initially registered in the cancer registry by the WHO grading system, which has been replaced in 2004 by the ICD-10 Tumor Lymph Node Metastasis (TNM) classification. Both are stored in DanProst.

All biopsies of the prostate were defined as being performed based on ultrasound and/or magnetic resonance imaging (MRI) using information from the National Patient Registry [17]. The National Patient Registry holds information on any activity within the Danish hospital systems. The register is based on the contacts of a specific person, it describes the condition and procedures performed for each contact. The register encompasses all the public, private, and psychiatric hospitals in Denmark. All SKS code registrations for ultrasound (UXUD) and MRI (UXMD) of the pelvic area that was related to a biopsy of the prostate (KTKE00, KKEB, 92,410, 91,640, or 91,320) were extracted from the National Patient Registry and based on occurrence within 31 days of the biopsy histology, it was considered an ultrasound biopsy, MRI biopsy or both.

The addition of PSA values was based on the PSA values available from DaPCaR, supplied with PSA levels available from the Danish Register of Laboratory Results for Research (LAB). LAB contains clinical biochemical and immunological laboratory results and has become available for research in 2018 with data from 2013 and onwards [18]. The register codes each of the measurements based on the NPU coding and is updated weekly, moreover historically electronically stored laboratory results are retrospectively added to the register when available. All PSA values in LAB for men in DanProst are extracted with the NPU codes NPU08* and NPU21*. To define the initial histology and diagnostic PSA values, first, the closest PSA value before the initial histology or diagnostic date was extracted. If the closest PSA value was more than 6 months before the initial histology or diagnosis and a PSA value after the initial histology date or diagnostic date was available within 3 months then the PSA value after was used. In men histologically diagnosed based on a radical prostatectomy specimen only PSA values before diagnosis were included.

Treatments are defined in several ways in DanProst to get a comprehensive picture of treatments related to prostate cancer. Radical prostatectomy is retrieved from the radical prostatectomy algorithm based on SNOMED coding (supplementary Fig. 7). From the National Patient Registry, information on curatively intended radiation therapy, androgen deprivation therapy, orchiectomy, chemotherapy, and novel hormonal therapy are extracted. Curatively intended radiation therapy of the prostate is defined by the occurrence of more than 20 BWGA, BWGC, or BWGE SKS codes within 61 days of each other. Androgen deprivation therapy is defined by the occurrence of BBHG32, BBHG33, BWHC3, BWHC52, ML02BX01, ML02BX02, ML02BX04, or ML02AE SKS codes. Orchiectomy is defined by the KKFC10, KKFC11, or KKFC13 SKS codes. Chemotherapy is defined by the BWHA263, BWHA208, ML01CH02, and ML01CH04 SKS codes related to a prostate cancer diagnosis. Novel hormonal therapy is defined by the BWHC50, BWHC51, ML02BB, or ML02BX03 SKS codes, with sub-specification for abiraterone possible. Lastly, for Active surveillance, watchful waiting and PSA observation is defined by the ZZ4252B, ZZ4252A, and ZZ4252 SKS codes, respectively, related to a prostate cancer diagnosis in the National Patient Registry. Unspecified observation was further defined by a PSA value in LAB if taken between 3 and 9 months after diagnosis. The algorithm to define the primary treatment is depicted in Supplementary Fig. 8.

SNOMED codes can be unspecific in their diagnosis, e.g. unspecified Gleason score or undefined adenocarcinoma. DaPCaR contains manually added detailed diagnostic information that was extracted from the written pathological report and the electronic health records. As such, if detailed information on the diagnostic conclusion was missing in DanProst of either the primary histology or prostate cancer diagnostic histology, detailed information in DaPCaR was copied to DanProst if the histological assessment date in DaPCaR was within 5 days of the histological assessment date in DanProst. The transfer of more detailed information in DaPCaR to DanProst was also performed for the procedure of diagnosis. When the diagnostic procedure was still unknown and if only one prostate SNOMED code was recorded in a histological assessment the tissue was regarded as taken by resection of the prostate and if more than one prostate SNOMED code was present for a single histological assessment, this prostate tissue was regarded as being taken by biopsy.

Lastly, DanProst has access to the Danish National Prescription Registry. The national prescription registry contains information on all drug prescriptions dispensed at Danish pharmacies since January 1, 1995 [19]. The registry, however, does not hold information on over-the-counter medications. Drugs are categorized according to the Anatomical Therapeutic Chemical (ATC) Classification System, a hierarchical classification system developed by the World Health Organization.

### Validation

Our translation algorithms were checked based on the assessments in DaPCaR in the period 2010–2016 as it was the most contemporary data available for comparison and because the registries have become more accurate over time in reporting. Data in DaPCaR are considered valid as all missing data has been manually assessed by two observers on an individual level. This contrasts with DanProst, which relies solely on algorithms for the translation of registry information.

## Results

An overview of all variables included in DanProst is depicted in Table 1. In short, there are 190,422 men in DanProst which is 37,215 (20%) more men than in the latest update of DaPCaR. The median age at primary histology is 70 years. Detailed conclusion of the primary histology is available for 36,287 (98%) of the newly added histological assessments after 2016. PSA values at primary histology are available for 63% (N = 119,807) in the entire cohort and in 94% (N = 35,130) of the men included after 2016. A total of 95,152 (50%) men are diagnosed with prostate cancer until 2021. Of these, 22,577 (12%) are new cases added on top of DaPCaR. Detailed information on diagnosis was available for 21,677 (96%) of the men diagnosed after 2016, without the addition of manual assessment. For the men diagnosed with prostate cancer, diagnostic PSA values are available for 73% (N = 69,429) in the entire cohort and for 99% (N = 22,278) of the men diagnosed after 2016. Most men are diagnosed by biopsy (N = 80,982; 85%) of which the majority was an ultrasound-guided biopsy (N = 63,751; 79%). The most frequently defined primary treatment was radical prostatectomy (N = 14,778; 19%), followed by curatively intended radiation therapy (N = 2,827; 14%).

**Table 1:** Characteristics of the Danish prostate registry (DanProst)

Histology	Variable		DanProst	After 2016
Primary histology	Number of men		190,422	37,215
	Age (Median, IQR)		70 (64–76)	70 (64–75)
	Topography	Prostate	173,941 (91%)	34,396 (92%)
		Prostate, bladder	7,748 (4%)	1,121 (3%)
		Prostate, vesicle	6,669 (4%)	941 (3%)
		Prostate, bladder, vesicle	1,746 (1%)	690 (2%)
		Other, unknown	318 (< 1%)	67 (0%)
	Procedure	Biopsy	119,015 (63%)	27,549 (74%)
		Resection	63,029 (33%)	8,267 (22%)
		Prostatectomy	3,704 (2%)	1,215 (3%)
		Autopsy	1,868 (1%)	131 (< 1%)
		Biopsy, resection	219 (< 1%)	37 (< 1%)
		Other, unknown	2,587 (1%)	16 (< 1%)
	Biopsy type	TRUS	91,890 (77%)	21,712 (79%)
		MRI	974 (1%)	495 (2%)
		TRUS + MRI	4,837 (4%)	3,216 (12%)
		Unknown	21,314 (18%)	2,126 (8%)
	Number of biopsies (median, IQR)		6 (6–10)	10 (6–12)
	Conclusion	GS < = 6	21,594 (11%)	4,393 (12%)
		GS 3 + 4	13,535 (7%)	5,266(14%)
		GS 4 + 3/5 + 2/2 + 5	7,166 (4%)	3,289 (9%)
		GS 5 + 3/3 + 5/4 + 4	10,367 (5%)	2,412 (7%)
		GS > 8	15,161 (8%)	3,775 (10%)
		GS 7 unspecified	5,231 (3%)	0
		Undefined AC	10,629 (6%)	809 (2%)
		NE	64 (< 1%)	18 (< 1%)
		SC	120 (< 1%)	16 (< 1%)
		Hyperplasia	54,656 (29%)	5,703 (15%)
		Dysplasia	1,056 (1%)	36 (< 1%)
		PIN	1,456 (1%)	789 (2%)
		Inflammation	8,857 (5%)	2,399 (6%)
		Benign	38,810 (20%)	8,191 (22%)
		Other/Unknown	1,670 (1%)	119 (< 1%)
	Number of positive biopsies (median, IQR)		1 (0–4)	2 (0–5)
	PSA (ng/ml) (median, IQR) Missing		7.7 (4.4–17)	7.4 (4.4–16)
			70,615 (37%)	2,085 (6%)
Diagnosis	Number of men		95,152	22,577
	Age (median, IQR)		71 (65–77)	71 (65–76)
	procedure	Biopsy	80,982 (85%)	20,910 (93%)
		Resection	11,385 (12%)	1,013 (5%)
		Biopsy + resection	199 (< 1%)	33 (< 1%)
		Radical prostatectomy	1,851 (2%)	592 (3%)
		Autopsy	663 (1%)	29 (< 1%)
		Other/Unknown	72 (< 1%)	0
	Biopsy type	TRUS	63,751 (79%)	16,157 (77%)
		MRI	1,199 (1%)	623 (3%)
		TRUS + MRI	4,923 (6%)	3,803 (18%)
		Unknown	11,109 (14%)	327 (2%)
	Number of biopsies (median, IQR)		6 (6–10)	10 (6–12)
	Conclusion	GS < = 6	25,674 (27%)	5,194 (23%)
		GS 3 + 4	15,540 (16%)	5,993 (27%)
		GS 4 + 3/5 + 2/2 + 5	8,106 (9%)	3,676 (16%)
		GS 5 + 3/3 + 5/4 + 4	11,547 (12%)	2,679 (12%)
		GS > 8	16,467 (17%)	4,098 (18%)
		GS 7 unspecified	6,328 (7%)	0
		Undefined AC	11,296 (12%)	900 (4%)
		NE	69 (< 1%)	18 (< 1%)
		SC	125 (< 1%)	19 (< 1%)
	Number of positive biopsies (median, IQR)		3 (1–6)	4 (2–6)
	PSA (ng/ml) (median, IQR) Missing		12 (6.6–36)	11 (6.3–31)
			25,723 (27%)	299 (1%)
	T-category	T1	21,190 (22%)	5,833 (26%)
		T2	20,467 (22%)	6,274 (28%)
		T3	19,018 (20%)	5,382 (24%)
		T4	2,434 (3%)	958 (4%)
		Other/unknown	32,043 (34%)	4,130 (18%)
	N-category	N0	20,995 (22%)	8,227 (36%)
		N1	5,064 (5%)	2,314 (10%)
		Other/unknown	69,093 (73%)	12,036 (53%)
	M-category	M0	33,923 (36%)	12,213 (54%)
		M1	9,096 (10%)	3,250 (14%)
		Other/unknown	52,133 (55%)	7,114 (32%)
	Primary treatment (% of known primary treatment)	Radical prostatectomy	14,778 (19%)	4,052 (20%)
		Radiation therapy	8,087 (11%)	2,827 (14%)
		GNRH targeted treatment	2,480 (3%)	1,259 (6%)
		Orchiectomy	3,230 (4%)	62 (< 1%)
		Novel hormone therapy	6,772 (9%)	2,299 (11%)
		Taxane treatment	1,859 (2%)	1,416 (7%)
		Active surveillance	6,330 (8%)	2,469 (12%)
		Watchful waiting	3,350 (4%)	896 (4%)
		Undefined observation	29,986 (39%)	5,176 (25%)
		Missing *(% of total men diagnosed)*	18,280 *(19%)*	2,121 (9%)
Radical prostatectomy	Number of men		18,464	5,629
	Gleason score	GS < = 6	2,839 (15%)	247 (4%)
		GS 3 + 4	7,357 (40%)	2,866 (51%)
		GS 4 + 3/5 + 2/2 + 5	3,276 (18%)	1,655 (29%)
		GS 5 + 3/3 + 5/4 + 4	877 (5%)	242 (4%)
		GS 9 + 10	991 (5%)	423 (8%)
		7 unspecific	2,153 (12%)	0
		Benign	46 (< 1%)	6 (< 1%)
		Hyperplasia/DY/PIN	319 (2%)	161 (3%)
		Other cancer	582 (3%)	27 (1%)
		Unknown	21 (< 1%)	< 5 (< 1%)
	Pathological T category	T0/Tx/Tis	1,105 (6%)	313 (6%)
		T1a + b	168 (1%)	11 (< 1%)
		T1c	18 (< 1%)	0
		Unspecified T1/Ta	35 (< 1%)	6 (< 1%)
		T2a + b	270 (2%)	43 (1%)
		T2c	9,493 (51%)	2,942 (52%)
		Unspecified T2	75 (< 1%)	28 (1%)
		T3 + 4	5,499 (30%)	2,068 (37%)
		Unknown	1,801 (10%)	221 (4%)
	Pathological N category	N0	885 (5%)	489 (9%)
		N1	3,609 (20%)	1,671 (30%)
		Nx	3,653 (20%)	1,337 (24%)
		Unknown	10,317 (56%)	2,132 (38%)
	Pathological M category	M0	26 (< 1%)	< 5 (< 1%)
		M1	12 (< 1%)	< 5 (< 1%)
		Mx	904 (5%)	< 5 (< 1%)
		Unknown	17,522 (95%)	5,653 (100%)
	Surgical margins	Positive	4,042 (22%)	1,315 (23%)
		Negative	12,070 (65%)	3,937 (70%)
		Unknown	2,352 (13%)	377 (7%)

**Abbreviations**: AC = Adenocarcinoma; NE = Neuro endocrine; SC = Small cell; DanProst = Danish Prostate Registry; GS = Gleason Score, IQR = Interquartile range; GNRH = Gonadotropin Releasing Hormone; TRUS = Transrectal ultrasound; MRI = Magnetic resonance imaging; PIN = Prostatic intraepithelial neoplasm; DY = Dysplasia

Comparison of DanProst with DaPCaR in the period from 2010 to 2016 showed that there was a high coherency in the translation of the procedures (> 99%), the conclusion of the primary histology (95%) and the conclusion of the diagnostic histology (94%) (Table 2).

**Table 2:** Comparison of the procedure and conclusion of the primary histology and the diagnostic conclusion between the Danish Prostate Registry (DanProst) and its predecessor the Danish Prostate Cancer Registry (DaPCaR)

Prostate-related procedure of the primary histology											
		DaPCaR									
		Biopsy	Resection	Prostatectomy	Autopsy	Biop. + Res.	Other	Unknown			
DanProst	Biopsy	40,070	27	< 5	0	< 5	13	25			
	Resection	32	10,810	< 5	< 5	0	55	9			
	Prostatectomy	47	21	9	< 5	0	11	0			
	Autopsy	< 5	0	0	234	0	0	0			
	Biop. + res.	24	< 5	0	0	7	0	0			
	Other	< 5	0	0	0	0	< 5	< 5			
	Unknown	0	0	0	0	0	0	0			
**Conclusion diagnosed in primary prostate histology**											
		DaPCaR									
		<= 6	3 + 4	4 + 3, 5 + 2, 2 + 5	>=8	7	AC	NE	SC		O/U
DanProst	<=6	6,408	189	13	8	91	9	0	0		83
	3 + 4	106	6,201	62	33	77	5	0	0		8
	4 + 3, 5 + 2, 2 + 5	5	201	2,795	36	104	0	0	0		< 5
	>=8	< 5	65	88	8,325	72	0	0	0		6
	7	0	0	0	0	377	0	0	0		< 5
	AC	< 5	0	< 5	< 5	25	182	0	0		1,298
	NE	0	0	0	0	0	7	6	0		6
	SC	0	0	0	0	0	0	< 5	19		12
	Hyperplasia	< 5	0	0	0	< 5	< 5	0	0		9,958
	Dysplasia	0	< 5	0	0	0	0	0	0		439
	PIN	< 5	0	0	0	0	0	0	0		665
	Inflammation	< 5	0	0	0	0	< 5	0	0		2,764
	Benign	5	< 5	0	< 5	0	< 5	0	0		14,762
	O/U	0	< 5	0	0	0	0	0	0		217
**Diagnostic conclusion**											
		DaPCaR									
		<= 6	3 + 4	4 + 3, 5 + 2, 2 + 5	>=8	7	AC	NE		SC	
DanProst	<=6	8,057	229	21	12	97	11	0		0	
	3 + 4	133	7,156	66	37	89	5	0		0	
	4 + 3, 5 + 2, 2 + 5	6	242	3,174	44	124	0	0		0	
	>=8	5	71	94	9,285	85	0	0		0	
	7	0	0	0	0	422	0	0		0	
	AC	263	106	33	40	42	240	0		0	
	NE	0	0	0	0	0	7	5		0	
	SC	0	0	0	0	0	0	< 5		22	

**Abbreviations**: AC = Adenocarcinoma; NE = Neuro endocrine; SC = Small cell; DanProst = Danish Prostate Registry; DaPCaR = Danish Prostate Cancer Registry; O/U = Other / Unknown

## Discussion and Perspectives

Here the methodology and early results for DanProst are presented. DanProst is an updated version of our previously published register named DaPCaR. Contrary to DaPCaR, DanProst is held in a central location that has direct access to other national registries. This allows for daily/weekly/monthly automatic updating based on the updates of the underlining registries. Looking at the above 97% completeness of the added information, the accuracy of the underlining registries is now sufficient to forgo manual assessment, as was the standard in DaPCaR. The flexibility of DanProst makes it possible to examine the current trends in clinical practice on a population-based level in all men undergoing either diagnostic workup for prostate cancer or having surgery performed in case of outlet obstruction. Furthermore, the new version of the database allows for a more updated follow-up and facilitates access to other registries enabling more extensive studies not only on prostate cancer but also on the trajectory of men with initial non-malignant diagnostic workup. This broadens the possibility of performing population-based studies of long-term consequences by gathering information from other sources.

The previous version of our registry, DaPCaR, has been criticized for its high number of missing PSA values, which has been the consequence of data not being stored in local laboratories. By adding information from the LAB, we have been able to significantly reduce the number of missing values in more contemporary men, which adds value to the data kept. Thus, in contemporary settings, the use of this important biomarker is possible. Access to biochemical information measured by the laboratories in Denmark also allows examination of other valuable endpoints in prostate cancer, such as biochemical recurrence and castration resistance [20, 21].

Another addition of DanProst is the use of any other treatment than radical prostatectomy, now both the primary treatment is available in the base dataset but also all consecutive treatments are obtainable for a longitudinal look at treatment trajectories in men with prostate cancer. To the best of our knowledge, longitudinal PSA and treatment data has not been available previously in a similar database. Moreover, all prescription-based treatments are available for men included in our dataset, allowing careful assessment of both side- and late effects related to treatments, as well as comorbidities of patients, and calculation of the economic aspects of prostate cancer treatments. The combination of the above-mentioned registries will give an exhaustive picture of the clinical practice of prostate cancer and prostate cancer biology and help us define the risks of important endpoints to refine the patient selection.

When adding more contemporary cases we observed a decrease in the proportion of men diagnosed with low-grade prostate cancer after 2016. Indicated by a higher proportion of men diagnosed with Gleason score 3 + 4 or above, and a higher proportion of men diagnosed with de novo metastatic disease after 2016. We hypothesize that this stage migration is due to the introduction of novel imaging such as MRI and PSMA PET. This hypothesis can now be tested using the centralized setup of DanProst.

DanProst is limited by its dependency on the quality of data entered in the included registries. However, it has previously been shown that the Danish registries hold highly valid data [22, 23]. Moreover, the accuracy of the underlining registry is consistently improving, as can be seen by the limited number of missing data in the most recent years. Other registries with high validity holding information on diagnosis and treatment exist [24, 25]. However, the main difference between the registry presented here and other registries, i.e. NoPCR and PCBaSE, is that the other registries are based on diagnostic codes from the cancer registry, whereas DanProst is based on all pathological assessments of all prostate tissue. This means that the men with histologically assessed lack of cancer in their prostate are the added value of DanPRost, a unique group of men that can function as a control group as well as a study group. Establishing similar registries as DanProst in other countries would be feasible. This requires that systematic registration of the results from pathological examinations in SNOMED codes is mandatory and that this personal health data is centrally stored. Furthermore, it may require special permission depending on national regulatory laws. Another limitation is the dependency on personally created algorithms to define variables. This implies a risk that a subset of the variables will be misclassified. Yet, as the coherency with our previous registry was high, we believe misclassification is limited. Nonetheless, our algorithms will be validated in future research by comparison of the algorithms with manual assessments. Lastly, the large proportion of undefined observation as primary treatment is a limitation. It is, however, mandatory to register all treatment procedures in the national patient registry. Thus, we believe in the accuracy of active treatment received after diagnosis. However, the registration of a specific surveillance strategy is not mandatory, and it is our clinical suspicion that the reporting of specified surveillance programs is inconsistent. Thus, future specification of the undefined observation is needed.

## Conclusion

DanProst is comparable to DaPCaR from 2010 to 2016 and adds a large set of new data that is highly detailed. The added value of DanProst is that the database is continuously updated as it is kept centrally with automatic integration of data from other national registries, allowing for contemporary nationwide analysis in men with histological assessment of the prostate from early clinical work-up to death.

### Electronic Supplementary Material

Below is the link to the electronic supplementary material.

Supplementary Material 1

## Data Availability

The data is available upon request to the authors and the Danish Data Protection authorities.
