# Supplementary material for: Danish Prostate Registry (DanProst) – an Updated Version of the Danish Prostate Cancer Registry, Methodology, and Early Results
Source: J Med Syst. 2023 Sep 13;47(1):98. doi: 10.1007/s10916-023-01991-8 (PMC10499673; doi:10.1007/s10916-023-01991-8)
Supplement: Supplementary file 1 — Supplementary Material 1 [file 10916_2023_1991_MOESM1_ESM.docx]

**Supplementary files**

Supplementary figure 1. Algorithm for translation of topography of the histological assessment based on SNOMED codes

**
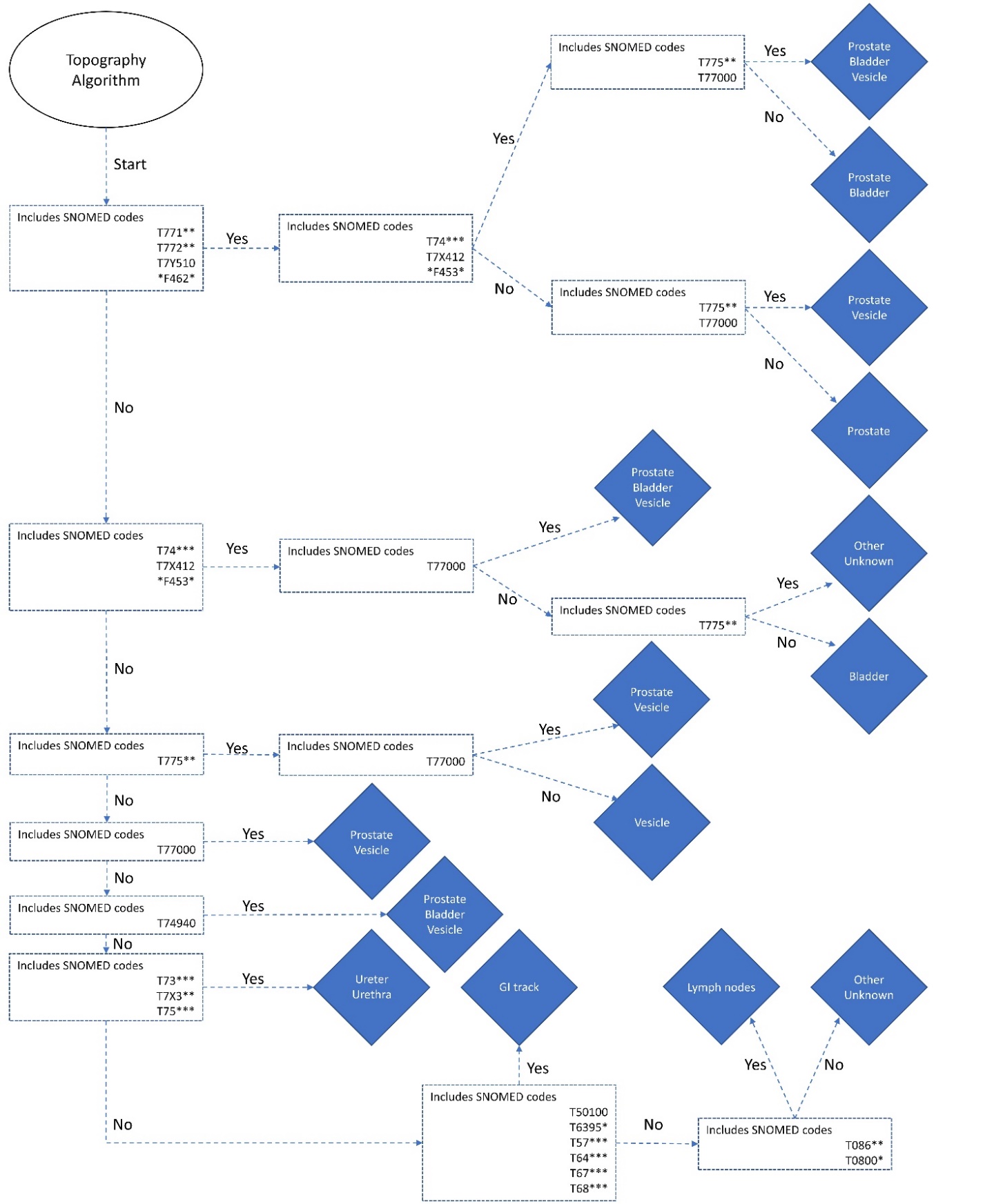
**

Supplementary figure 2. Algorithm for translation of procedure that was used to obtain the histological specimen based on SNOMED codes

**
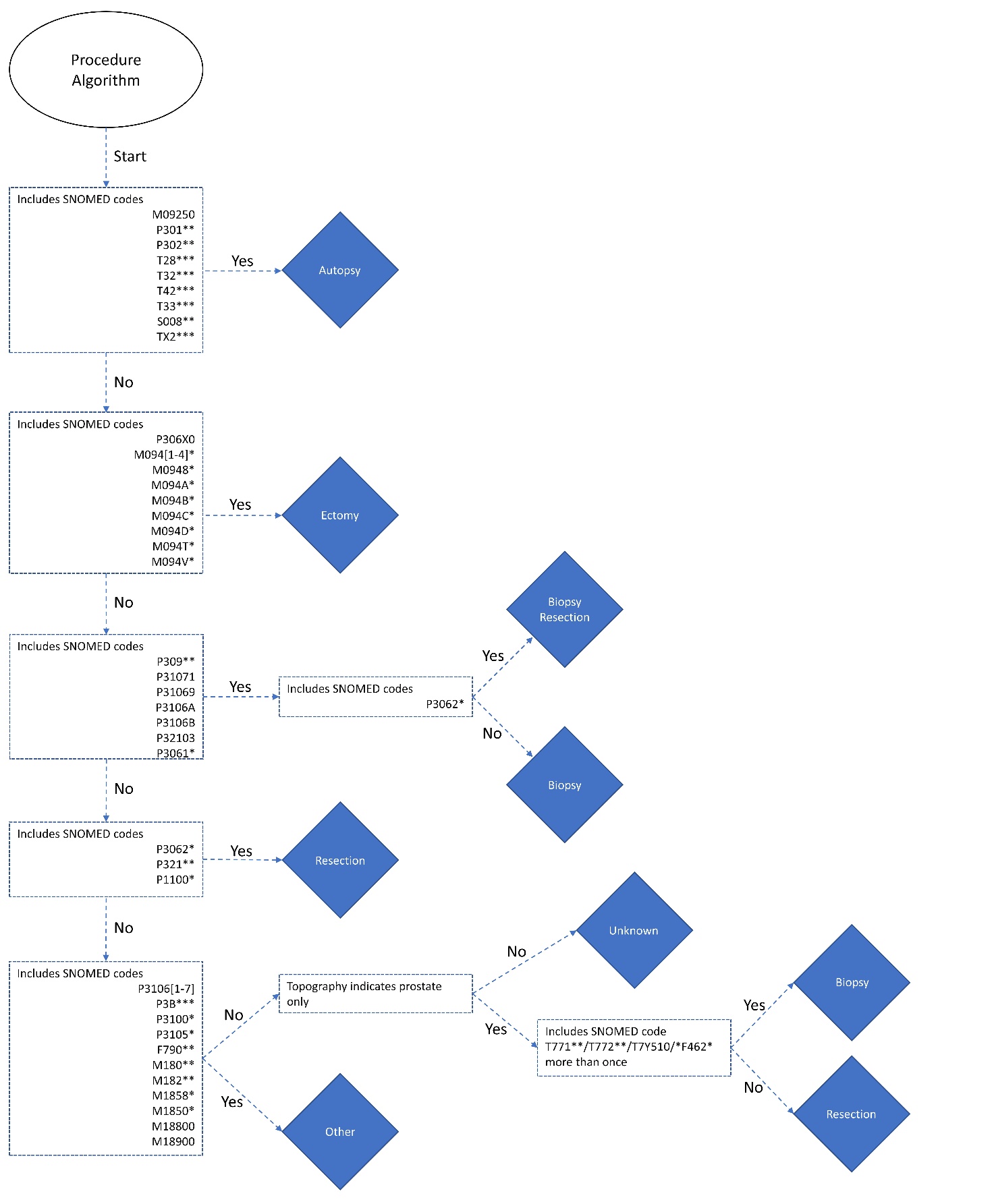
**

Supplementary figure 3. Algorithm for translation of the histological diagnosis based on SNOMED codes

**
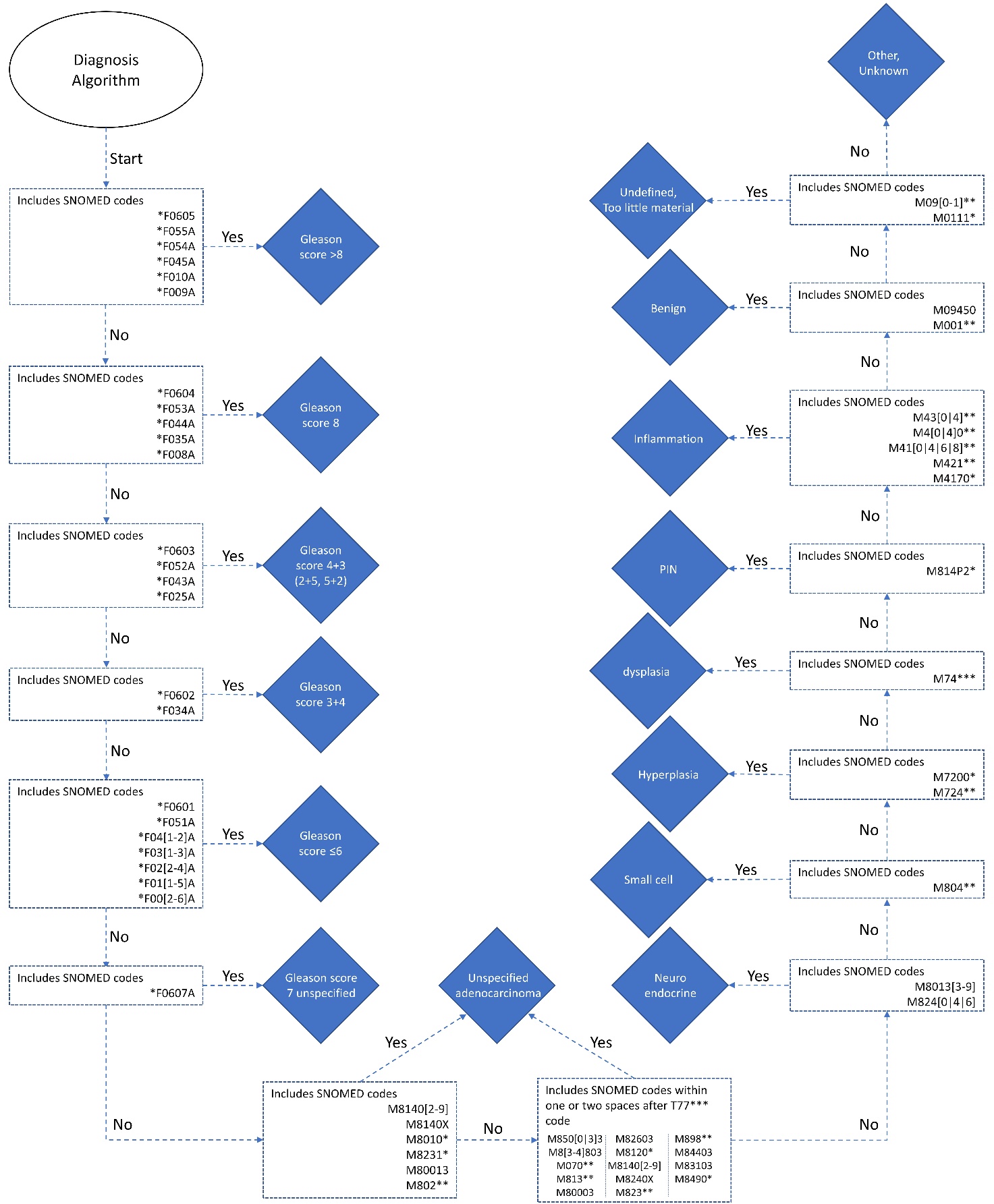
**

Supplementary figure 4. Algorithm for translation of pathological tumor classification based on SNOMED codes

**
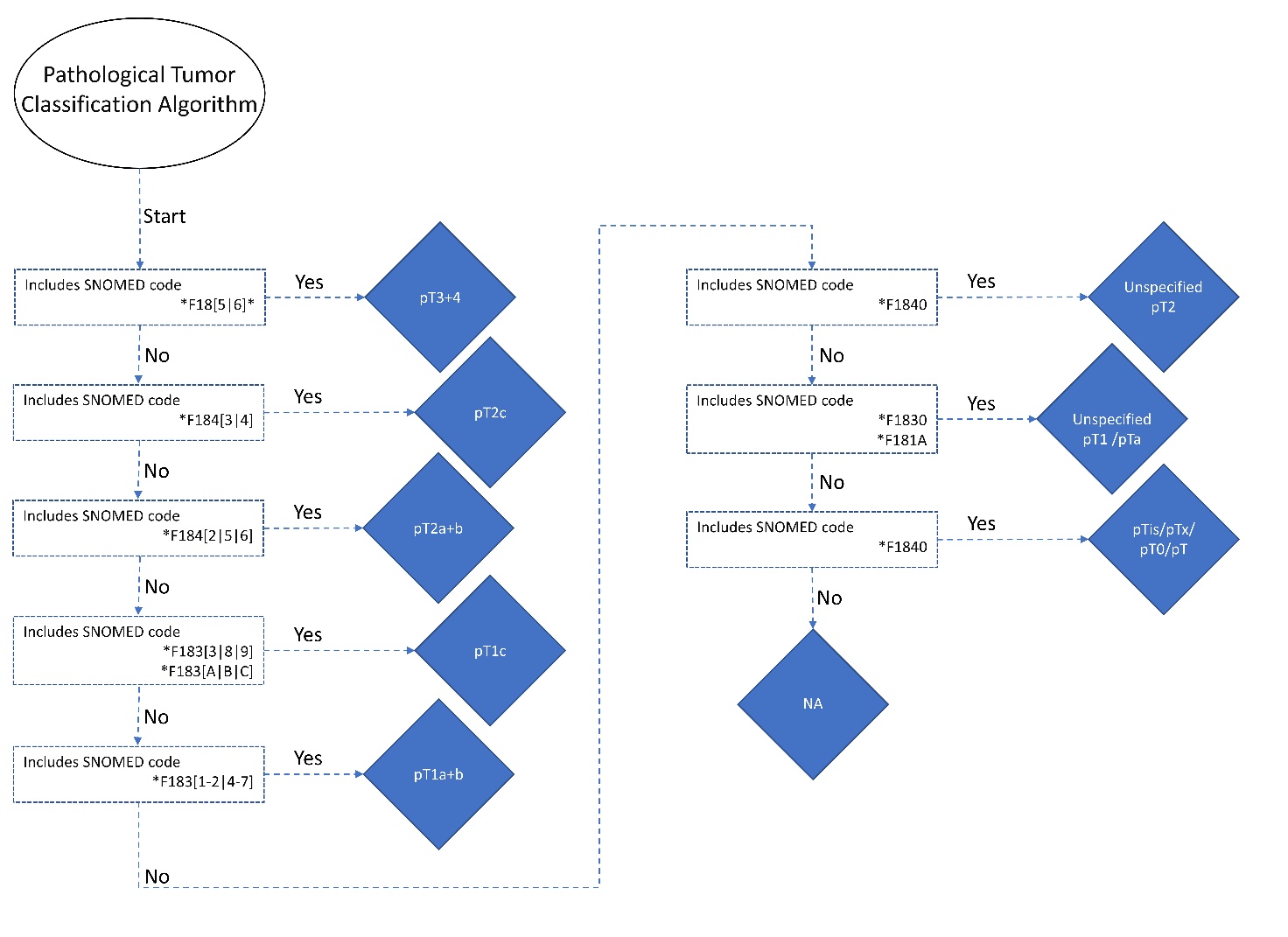
**

Supplementary figure 5. Algorithm for translation of pathological lymph node classification based on SNOMED codes

**
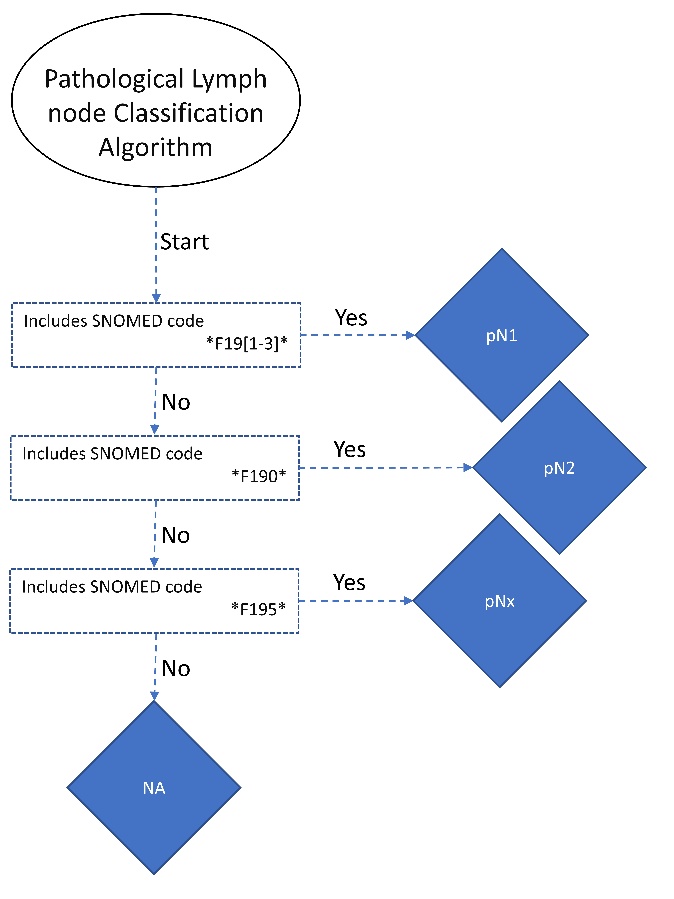
**

Supplementary figure 6. Algorithm for translation of pathological metastasis classification based on SNOMED codes

**
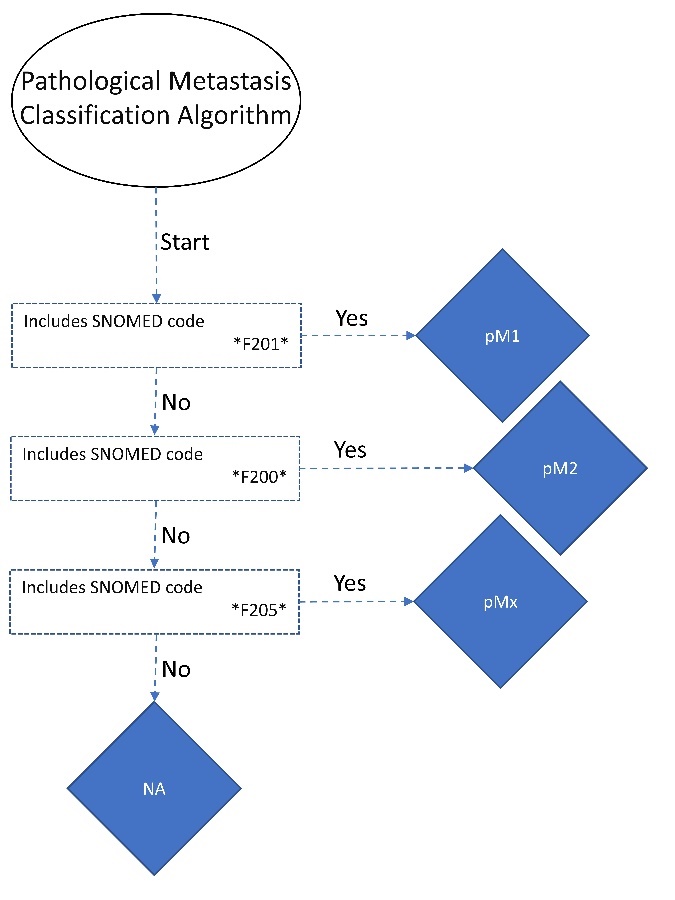
**

**
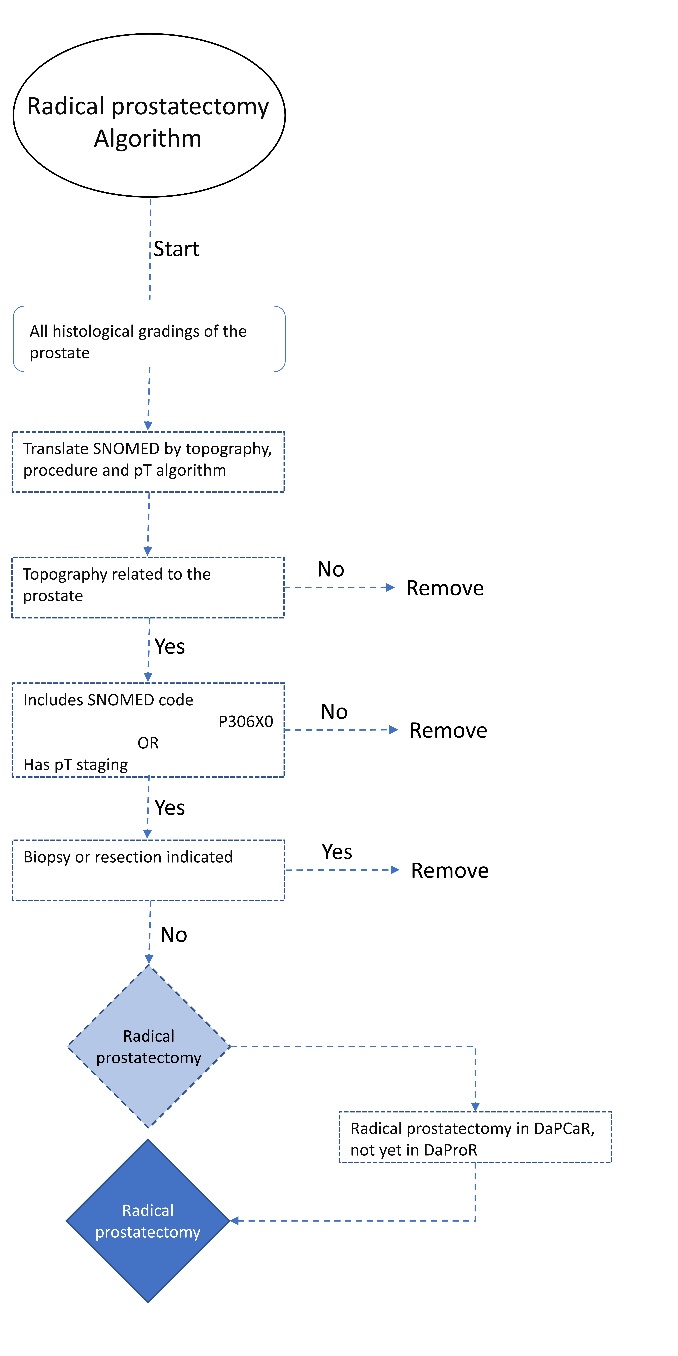
**Supplementary figure 7. Algorithm for translation of radical prostatectomy based on SNOMED codes.

Supplementary figure 8. Algorithm for definition of primary treatment following prostate cancer diagnosis based on SNOMED codes, national patient registry and laboratory registry.

**
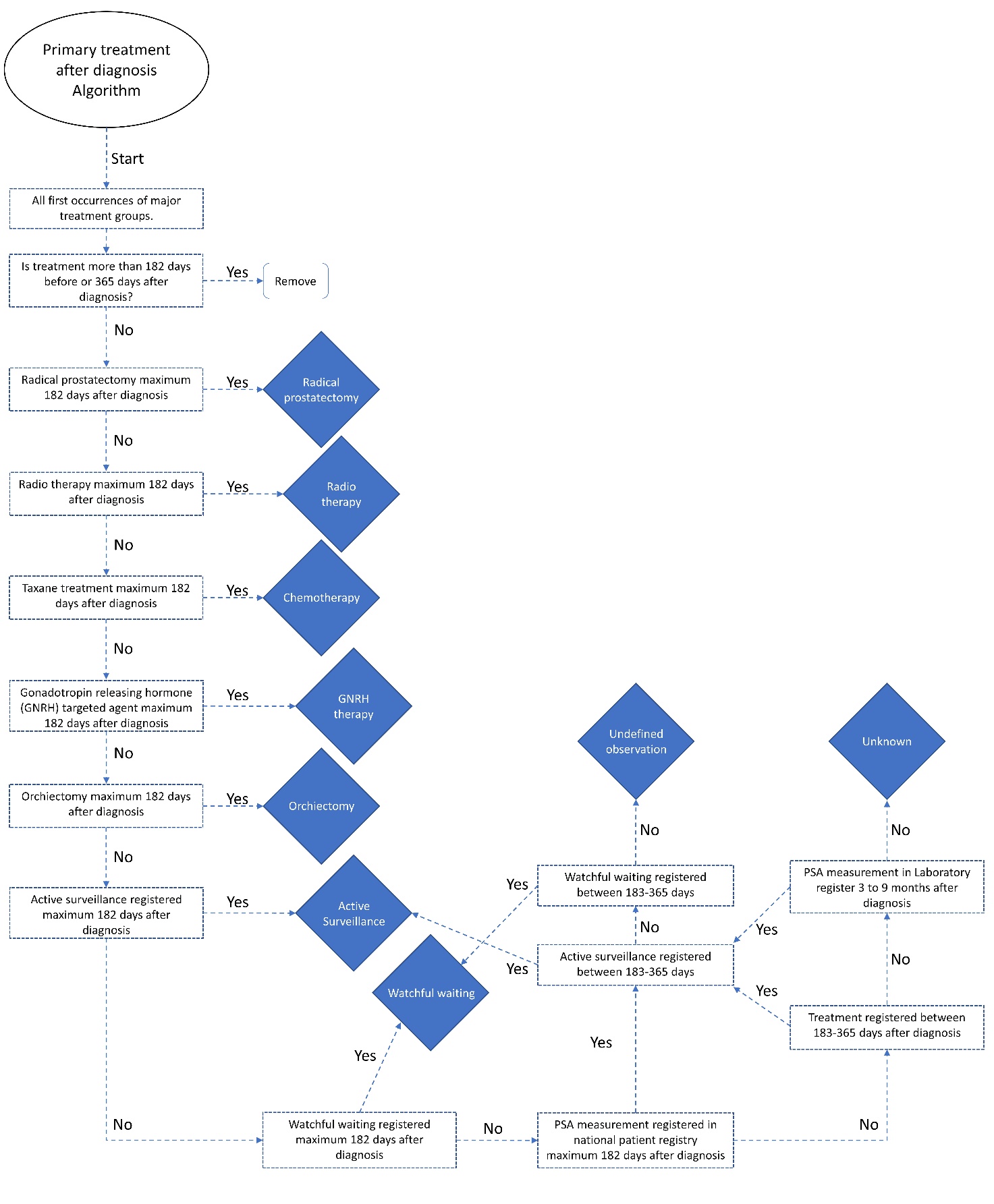
**
